# Progressive Micrographia Shown in Horizontal, but not Vertical, Writing in Parkinson’s Disease

**DOI:** 10.3233/BEN-120285

**Published:** 2013

**Authors:** Hui-Ing Ma, Wen-Juh Hwang, Shao-Hsia Chang, Tsui-Ying Wang

**Affiliations:** ^1^Department of Occupational Therapy and Institute of Allied Health SciencesMedical CollegeNational Cheng Kung UniversityTainanTaiwan; ^2^Department of NeurologyMedical CollegeNational Cheng Kung UniversityTainanTaiwan; ^3^Department of Occupational TherapyI-Shou UniversityKaohsiungTaiwan

**Keywords:** Handwriting, micrographia, motor skill, Parkinson’s disease

## Abstract

All published studies on micrographia, a diminution of letter size, examine handwriting in the horizontal direction. Writing horizontally typically requires increased wrist extension as handwriting progresses from left to right. Chinese characters, however, can be written not only horizontally from left to right, but also vertically from top to bottom. We examined the effect of handwriting direction on character size and stroke length. Fifteen participants with Parkinson’s disease (PD) and 15 age-matched controls wrote the same Chinese characters both horizontally and vertically. Handwriting performance was recorded with a digitizing tablet, and a custom-written computer program was used to provide objective data about character size and stroke length. The PD group had a linear decrease in overall character size and horizontal strokes along the writing sequence in the horizontal direction, but not in the vertical direction. The controls had shorter horizontal strokes in the horizontal than the vertical direction, but there was no progressive shortening of stroke length along the writing sequence. The results suggest that traditionally reported progressive micrographia in horizontal writing may not be generalizable to vertical writing. The observed decrease of handwriting size in the horizontal direction suggests that micrographia in PD may be associated with wrist extension. For clinical implications, patients may mitigate their micrographia by changing handwriting direction.

## 1. Introduction

Patients with Parkinson's disease (PD) experience handwriting deterioration even early in the disease [1]. Their handwriting size diminishes, referred to as micrographia, which often manifests in two forms: consistent micrographia (a constant reduction in size) and progressive micrographia (a successive reduction in size as the writing progresses) [2]. Because micrographia is easy to observe and of functional importance, assessing handwriting for signs of micrographia has been widely used in research and clinics to monitor the progress of the disease and the effect of medical interventions [3–5].

Factors contributing to micrographia in PD are still under investigation. Studies have suggested that it may be associated with increased cognitive demands [6,7], poor coordination between simultaneous wrist and finger movements [8,9], and wrist extension stiffness [7]. All the published studies on micrographia, however, examined handwriting in the horizontal direction and analyzed samples primarily from Western countries [10–12].

It should be noted that handwriting in some countries runs in different directions. For some countries in Eastern Asia (e.g., Japan, Korea, and Taiwan), handwriting

ISSN 0953-4180/13/$27.50 © 2013 – IOS Press and the authors. All rights reserved may run horizontally from left to right as well as vertically from top to bottom. The different directions require different joint coordination patterns, and writing horizontally (for a right-handed person) requires more wrist extension [7] than does writing vertically. Therefore, the micrographia reported in horizontal writing may not be generalizable to vertically written characters.

The purpose of this study was to fill this gap in knowledge by examining the differences between horizontal and vertical Chinese handwriting in Taiwanese patients with PD. Specifically, we were interested in the trend of handwriting size along the writing sequence to know whether progressive micrographia reported in horizontal writing would also be evident in vertical writing. The information should shed light on factors contributing to micrographia, and open up possibilities for clinical applications.

## 2. Methods

### 2.1. Participants

We enrolled a convenience sample composed of 15 participants with idiopathic PD (5 women, 10 men; mean age: 65.5 ± 7.0 years old) and 15 age-matched controls (10 women, 5 men; mean age: 61.8 ± 8.6). Patients with PD were included if they were at Hoehn and Yahr stage 1 to 3, right-handed, literate, and able to write. Patients were excluded if they had other neurological or musculoskeletal disorders affecting writing movement. The PD group had a mean disease duration of 4.4 ± 2.3 years. Their Hoehn and Yahr stage ranged from 1 to 3 with a median of 2. Healthy right-handed, age-matched controls were also recruited. Our university hospital Institutional Review Board approved this study, and all participants gave informed consent.

### 2.2. Design and procedures

A counterbalanced repeated-measures design was used, in which participants were randomly assigned to writing in either the horizontal or the vertical direction first, and the other direction second. As a result, 8 participants with PD and 8 controls wrote in the horizontal direction first, and the other 7 participants with PD and 7 controls wrote in the vertical direction first.

To perform the experimental tasks, each participant was asked to sit at a table on which 72 × 29.8-cm sheets of white paper were placed on a digitizing tablet (Intuos3 PTZ-930; Wacom Co., Japan). The digitizer was positioned perpendicular to the forearm. All participants performed, at their own pace, two test trials in each direction. Each participant wrote four instances of "正" at a comfortable size and speed on the unruled paper, with its long axis aligned with the horizontal axis of the tablet. The test trials were used for analysis of handwriting size.

We used the Chinese character "Zheng" (正), a common Chinese character that means *proper, right, straight, fair, positive* [5]. It has five distinct strokes, written from top to bottom and from left to right (Fig. 1). Zheng was chosen for several reasons. First, this character is also used when counting to five (like 卌 in the west), so it is neither unusual nor awkward for people to write a series of Zhengs. In addition, its purely horizontal and vertical strokes, rather than oblique or circular (loops) strokes, allow us to clearly classify the types of movements required to complete them. Finally, this character has a well-defined overall square shape and, therefore, the size of each character can easily be measured.

### 2.3. Instrument

An ink pen (Intuos3 ZP-130; Wacom) was used with the digitizing tablet to record the handwriting movement. The tablet (maximum frequency: 200 Hz; resolution: 1000 lines per cm; spatial accuracy: 0.015 cm; temporal accuracy: 1 ms) samples the X (horizontal) and Y (vertical) positions of the pen tip as well as the axial pen force. The data were stored for off-line analysis, and a technical computing program (Matlab 7.6; The MathWorks, Natick, MA, USA) was used to derive the data. *Character size* refers to the size of "正" and was defined as the area of the quadrilateral outlined by the top, bottom, left, and right margins of each character. For the *stroke length*, we averaged the length of the writing trace for strokes 1 and 5 (the long top and bottom horizontal strokes), and measured the length of stroke 2 (the long central vertical stroke).

### 2.4. Data analysis

To examine any differences of handwriting trend between PD and control groups, we used a general linear model (GLM) for repeated measures to perform 2-way mixed analysis of covariance (ANCOVA) for each handwriting direction [13,14]. The independent variables were *group* (between factor: PD vs. controls) and *sequence* (within factor: $2^{nd}$ vs. $3^{rd}$ vs. $4^{th}$ character).

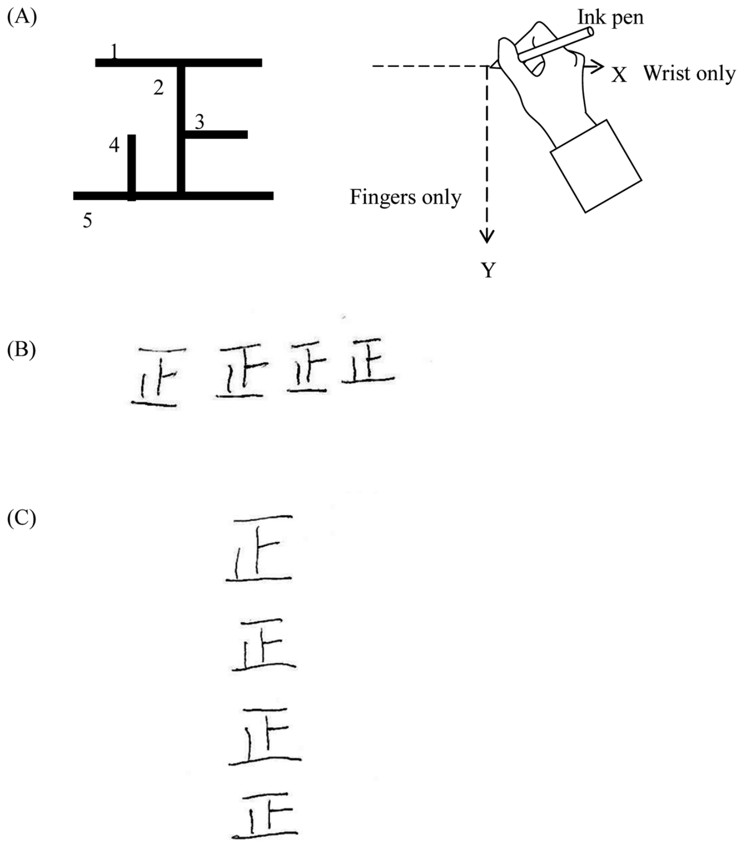

Fig. 1. (A) An illustration of the stroke order for writing "正" and movements for writing the strokes (wrist extension for the horizontal strokes and finger flexion for the vertical strokes). (B) A sample of horizontal writing from a person with Parkinson's disease (PD#01). (C) A sample of vertical writing from a person with Parkinson's disease (PD#01).

The size of the $1^{st}$ character was used as the covariate to control for the variability in initial handwriting due to various factors, such as personal habit [2]. To examine any differences of handwriting trend between horizontal and vertical writing, repeated measures ANCOVA was used for each group. Handwriting direction (within factor: horizontal vs. vertical) and sequence were used as independent variables and the $1^{st}$ character as the covariate. Analyses were performed using SPSS Version 17 (SPSS, Inc., Chicago, IL, USA).

## 3. Results

### 3.1. Between group differences

The results of the 2-way mixed ANCOVA on character size indicated significant *group* ($p = 0.041$), *sequence* ($p = 0.022$), and *group* × *sequence* ($p = 0.007$) effects in the horizontal direction. When writing horizontally, the PD group had decreased character size along the writing sequence, while the control group did not (Fig. 2). For the vertical direction, no significant main or interaction effect was found.

### 3.2. Within group differences

The results of the repeated measures ANCOVA on character size indicated a significant *direction* × *sequence* effect for the PD group ($p = 0.043$). Inspection of the data showed that while the PD group had linear decreases along the writing sequence in the horizontal direction, the decreases occurred from the $1^{st}$ to $3^{rd}$ character, but that there was an increase from the $3^{rd}$ to $4^{th}$ character in the vertical direction. The control group had no significant main or interaction effect.

Because the character size was dependent on the length of long horizontal strokes (strokes 1 and 5) and the long vertical stroke (stroke 2), we also examined the effect of handwriting direction on these stroke lengths.

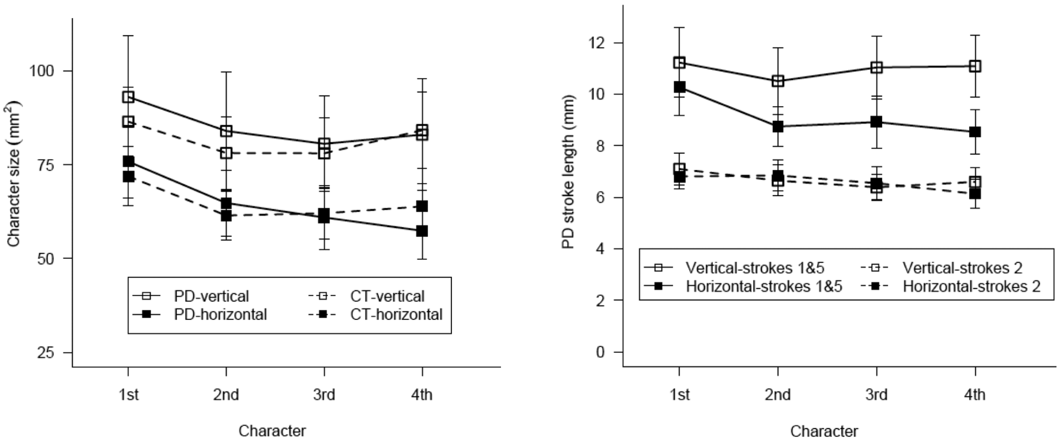

(A) (B)

Fig. 2. (A) Character size (mean ± standard error) of Parkinson's disease and control groups in the horizontal and vertical directions. (B) Stroke length (mean ± standard error) of the PD group in the horizontal and vertical directions. CT = controls; PD = Parkinson's disease.

The results of ANCOVA on horizontal stroke length (average of strokes 1 and 5) showed significant *direction* ($p = 0.002$), *sequence* ($p = 0.045$), and *direction* × *sequence* ($p = 0.006$) effects for the PD group. Inspection of the data also showed that the PD group shortened their horizontal strokes along the writing sequence in the horizontal direction, but not in the vertical direction (Fig. 2). For the controls, there was only a significant *direction* effect ($p = 0.014$). The controls had shorter strokes 1 and 5 in the horizontal than in the vertical direction. There was no significant main or interaction effect on the length of the vertical stroke (stroke 2) for the PD or control group.

## 4. Discussion

By using the unique characteristics of Chinese characters that can be written from left to right as well as from top to bottom, we provide important evidence that progressive micrographia in patients with PD, which was reported in horizontal writing, is not evident in vertical writing. The progressive reduction of character size was mainly due to the shortening of the horizontal stroke, rather than the vertical stroke. In comparison, the only effect seen in the control group was shorter horizontal strokes, regardless of sequence, in the horizontal than in the vertical direction.

Micrographia has been associated with increased cognitive demands [6,7], poor coordination between simultaneous wrist and finger movements [8,9], and wrist extension stiffness [7]. In this study, the cognitive demands in both experimental writing tasks were similar, because Taiwanese people are familiar with both vertical and horizontal writing. In addition, the need to coordinate simultaneous wrist and finger movements are minimal, because the distinct horizontal and vertical strokes in "Zheng" require pure wrist extension and finger flexion movement, respectively. Therefore, our results point to the role of wrist extension stiffness in progressive micrographia.

For a right-handed person, as handwriting progresses from left to right across the writing surface, more wrist extension is typically required [9]. While healthy people can voluntarily relax their wrist muscles when writing, patients with PD manifest a constant background muscle activity and muscle tension due to rigidity [15]. Moreover, by using quantitative measures of torque resistance, researchers [16,17] demonstrated the asymmetric feature of rigidity and found that rigidity is more pronounced during wrist extension than flexion movement in patients with PD. Therefore, the greater resistance in further extending their wrist results in shorter horizontal strokes. Our results support the notion by showing that patients had significant reductions in the length of horizontal strokes that require wrist extension, but not in vertical strokes that demand finger flexion. Our results are also consistent with a report [7] that showed a non-significant effect of wrist extension on the handwriting size of cursive "*llll*" that required a combination of finger and wrist movements.

By using the $1^{st}$ character as the covariate, we found that the PD group decreased their character size along the writing sequence when writing horizontally. The results suggest that even though patients with PD may begin by writing big characters, they are unable to maintain their character size and thus reduce the size gradually when writing from left to right. In our study, we did not use handwriting difficulty as an inclusion criterion. The results of significant *group* $\times$ *sequence* effect suggest that analyzing the trend of handwriting size along the writing sequence is a sensitive measure for detecting handwriting impairments in patients with PD.

This study has some limitations. First, we measured only character size and stroke length. Other writing kinematics such as amplitude of maximum velocity and the number of movement units should be examined in future research to provide information on the force and smoothness of handwriting. Second, we inferred, rather than directly measured, joint movements in writing. It is worthwhile for future research to directly examine the joint movements. Third, we had the paper aligned horizontally in both conditions. Thus in the vertical handwriting direction relative to the horizontal direction, there was a proportionally more constrained paper area to finish the four characters. It is unknown whether the more constrained area in the vertical condition would have helped the performance of the PD group. Future research can either examine the effect of paper alignment or else control this factor by providing a square sheet of paper. Fourth, although we found significant effects of handwriting direction on character size and horizontal strokes, the small sample size in this study may limit the power to detect some moderate or small effects of handwriting direction. Future research with a larger sample size will be important for detecting potential differences that may provide alternative explanations for the effect of handwriting direction observed in this study. Finally, investigating the handwriting performance of patients with PD in different contexts and cultures may help further clarify the mechanism of micrographia. For example, in some written languages, writing is from right to left, which provides an opportunity to examine the effect of different joint movement patterns.

One clinical implication of our findings is that evaluating patients' handwriting in both horizontal and vertical directions (when culturally appropriate) provides insight into the role of wrist extension in their handwriting performance. A change in handwriting direction to decrease the need of wrist extension may be a feasible strategy for patients with PD to improve their handwriting. Alternatively, patients may adjust their arm position or exercise their wrist to decrease stiffness while writing. Additional research, however, is needed to verify the effectiveness of these strategies.

## Acknowledgments

This project was supported by grant NSC 94-2314-B-006-031 from the National Science Council, Taiwan. The authors would like to thank Dr. Paul Saunders for helpful discussions and editing advice, research assistant Mr. Chung-Ying Lin for the statistical assistance, and the anonymous reviewers for their valuable comments and suggestions to improve the manuscript.
